# Insolation-paced sea level and sediment flux during the early Pleistocene in Southeast Asia

**DOI:** 10.1038/s41598-021-96372-x

**Published:** 2021-08-18

**Authors:** Romain Vaucher, Shahin E. Dashtgard, Chorng-Shern Horng, Christian Zeeden, Antoine Dillinger, Yu-Yen Pan, Romy A. Setiaji, Wen-Rong Chi, Ludvig Löwemark

**Affiliations:** 1 Applied Research in Ichnology and Sedimentology (ARISE) Group, Department of Earth Sciences, Simon Fraser University, Burnaby, Canada; 2 Institute of Earth Sciences, Academia Sinica, Taipei, Taiwan; 3 LIAG—Leibniz Institute for Applied Geophysics, Geozentrum Hannover, Hannover, Germany; 4 Centre for Natural Hazards Research, Department of Earth Sciences, Simon Fraser University, Burnaby, Canada; 5 Department of Geosciences, National Taiwan University, Taipei, Taiwan; 6 Department of Earth Sciences, National Central University, Taoyuan, Taiwan; 7 Department of Earth Sciences, National Cheng-Kung University, Tainan, Taiwan

**Keywords:** Geology, Palaeomagnetism, Sedimentology, Palaeoclimate, Natural hazards

## Abstract

Global marine archives from the early Pleistocene indicate that glacial-interglacial cycles, and their corresponding sea-level cycles, have predominantly a periodicity of ~ 41 kyrs driven by Earth’s obliquity. Here, we present a clastic shallow-marine record from the early Pleistocene in Southeast Asia (Cholan Formation, Taiwan). The studied strata comprise stacked cyclic successions deposited in offshore to nearshore environments in the paleo-Taiwan Strait. The stratigraphy was compared to both a δ^18^O isotope record of benthic foraminifera and orbital parameters driving insolation at the time of deposition. Analyses indicate a strong correlation between depositional cycles and Northern Hemisphere summer insolation, which is precession-dominated with an obliquity component. Our results represent geological evidence of precession-dominated sea-level fluctuations during the early Pleistocene, independent of a global ice-volume proxy. Preservation of this signal is possible due to the high-accommodation creation and high-sedimentation rate in the basin enhancing the completeness of the stratigraphic record.

## Introduction

Climate oscillations are controlled by variations in Earth’s orbital and astronomical motions (i.e., precession, obliquity, eccentricity), which modulate changes in insolation received by the atmosphere. These oscillations manifest as periods of colder and warmer climate that affect the growth and decay of ice sheets and produce quasi-cyclic sea-level fluctuations^1,2,3,4,5,6^. Variation in obliquity (axial tilt relative to the orbital plane) acts on the latitudinal distribution of insolation, and has a dominant impact on insolation received at high latitudes. Obliquity cycles have a periodicity of ~ 41 kyrs, and dominated climate cycles globally during the early Pleistocene. Precession (wobble of the Earth around its tilted axis) is modulated by eccentricity (shape of Earth’s orbit around the sun) and drives the seasonal distribution of insolation between the southern and northern hemispheres. Precession cycles have a dominant frequency of ~ 21 kyrs^7,8^. The modulation of atmospheric moisture and ice accumulation at high latitudes is mostly linked to obliquity, while precession controls the intensity of the summer insolation and affects hydrological cycles especially at low latitudes^1,5,9,10,11^.

Shallow-marine settings are directly affected by long- and short-term climatic variations (e.g., sea level changes, extreme weather events) and their preserved stratigraphic architecture is controlled primarily by variations in sea level, sediment supply and subsidence^12^. These latter factors control the preservation and scale of depositional cycles in sedimentary basins^13,14^. Accurately determining the manifestations of climate oscillations in the sedimentary record requires matching depositional cycles to climatic models^15,16,17^. However, the identification of climate oscillations in shallow-marine environments is not straightforward because the stratigraphic record commonly has a low temporal completeness^18^. Indeed, sediment bypass and reworking are common in shallow-marine environments. High temporal completeness of the stratigraphic record is required to accurately determine the controls on cyclicity in the sedimentary record, including shallow-marine strata. Such critical conditions likely occur in sedimentary basins with young sedimentary strata that accumulated under high rates of both sediment accumulation and accommodation creation as these settings promote the completeness of their record.

**Figure 1:**
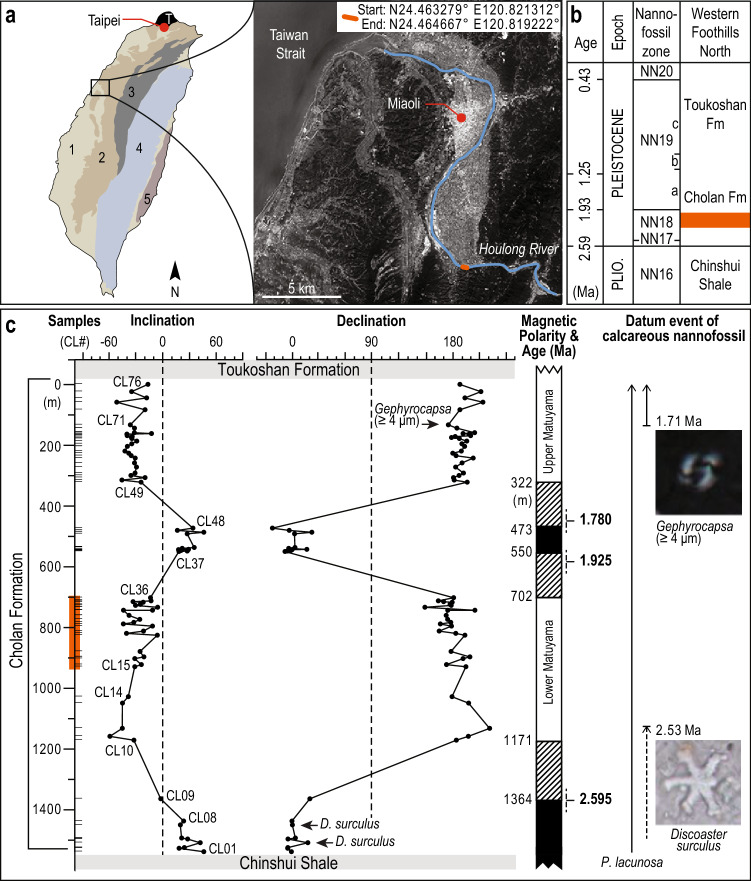
Geographic and stratigraphic framework. (**a**) Simplified geological map of Taiwan: 1—Coastal Plain; 2—Western Foothills; 3—Hsuehshan Range; 4—Central Range; 5—Coastal Range; T—Tatun volcano group (map modified from Lin and Chen^28^). The studied section stretches along the Houlong River. The satellite map is from Google Earth. (**b**) The chronostratigraphic chart of the northern part of the Western Foothills ^22^. (**c**) The magneto-biostratigraphic framework developed in this study places the studied section within the lower Matuyama reversed epoch close to the lower limit of the Olduvai normal polarity subchron. The intense vegetation cover on the outcrop presently prevents sampling near the polarity boundaries (hatched intervals in the Magnetic Polarity & Age (Ma) column). The geomagnetic polarity time scale^29^ and the datum events of index calcareous nannofossils^30^ are shown. Ol: Olduvai. Orange lines denote the studied stratigraphic interval. The illustration was made using Adobe Illustrator CS6 (https://www.adobe.com/).

**Figure 2:**
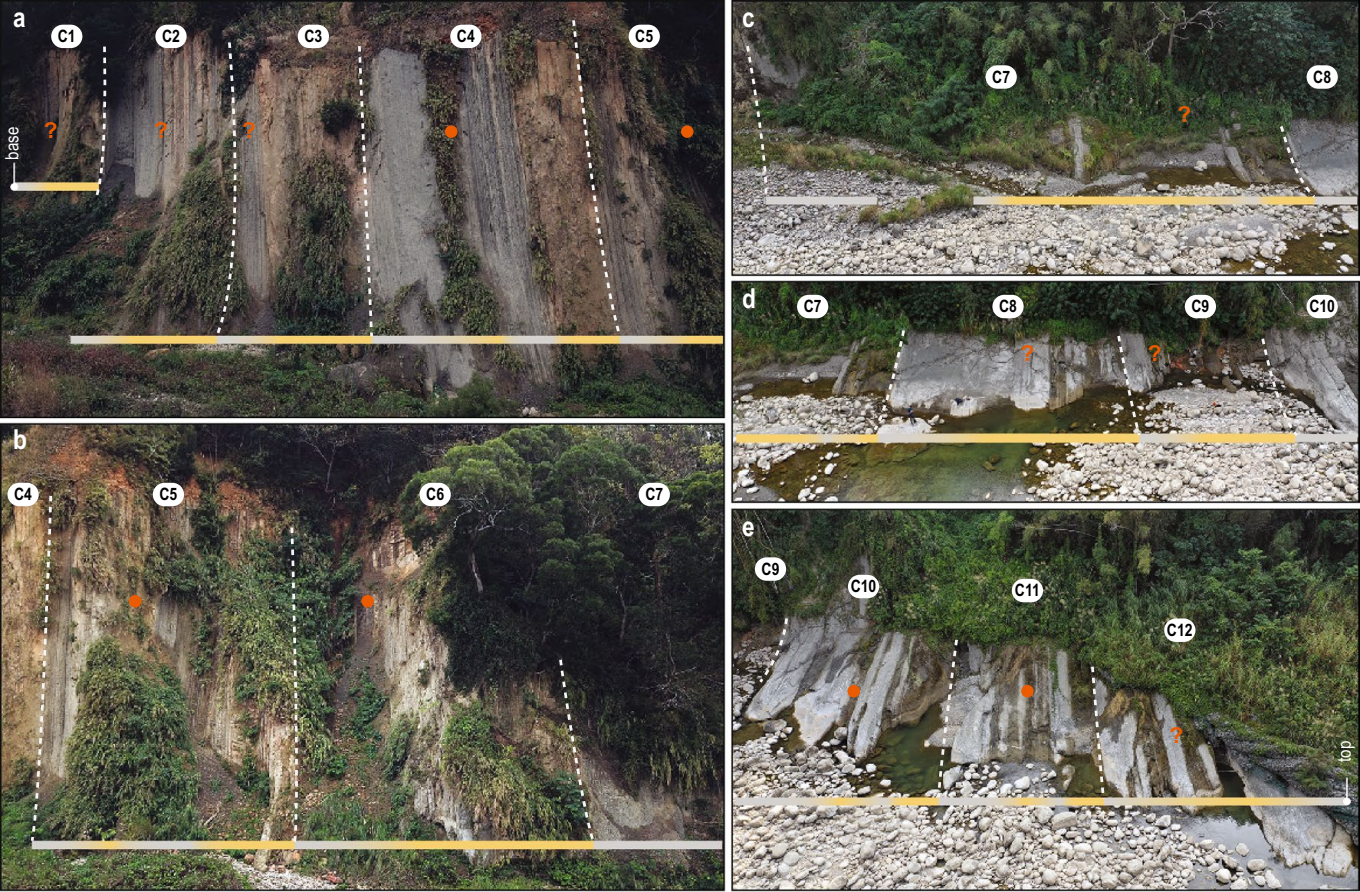
Drone footage of the lower Cholan Fm along the Houlong River (Fig. 1). Twelve cycles (C1–C12) are defined for the 241 m thick section, evolving from mudstone-prone to sandstone-prone sedimentary facies. Color scale bar: light gray: mudstone-prone, yellowish: sandstone-prone. Orange dots (and question marks) mark the climax of interactions between tropical cyclones and the monsoon, which are expressed as sandstone-prone heterolithics intervals. The illustration was made using Adobe Photoshop CS6 and Adobe Illustrator CS6 (https://www.adobe.com/).

Plio-Pleistocene shallow-marine sedimentary strata in the Western Foreland Basin (WFB) of Taiwan accumulated under high rates of sedimentation and accommodation creation, suggesting that the record should have relatively high temporal completeness^19,20,21,22,23,24,25^. In the lower Pleistocene Cholan Formation (Fm; Fig. 1), depositional cycles are expressed prominently (Fig. 2) and the relatively young age of these shallow-marine strata enables comparison with depositional processes in the modern Taiwan Strait^26,27^.

Herein, we combined facies analysis of a 241 m-thick section from the lower Cholan Fm with magneto-biostratigraphy to constrain time and assess controls on depositional cyclicity. Our data are then compared to reference curves to determine the relation between depositional cyclicity and orbitally forced changes in insolation. We then discuss which mechanisms influenced sea level and sediment supply during deposition. Finally, we discuss the utilization of shallow-marine strata as climate archives, and the parameters that are required to ensure high temporal completeness.

## Geological setting

Taiwan is situated at the convergent boundary between the Eurasian Plate and Philippines Sea Plate. The onset of the Taiwan Orogeny that formed the Taiwan Strait and the WFB started around 6.5 Ma and continues today^23,24^. The Western Foothills—part of the WFB (Fig. 1a)—form a fold-and-thrust belt that exposes the most recently deposited sedimentary successions (from Late Oligocene to modern) that accumulated successively during passive margin, rift, post-rift, and foreland basin stages^23,24,25,31^. The fast-growing orogen is responsible for high rates of accommodation creation in the WFB, and both the orogen and tropical climate contribute to the high sedimentation rate^20,21,32,33,34,35^. The lower Pleistocene Cholan Fm, exposed in the Western Foothills, comprises dominantly heterolithic strata, and previous interpretations suggest deposition was influenced by wave-, tide- and/or river-processes in relatively shallow-marine environments ranging from offshore (> 15 m water depth) to the nearshore (< 15 m water depth)^36,37,38^. The Cholan Fm overlies marine mudstone of the Chinshui Shale (late Pliocene) and is overlain by terrestrial conglomerate of the Toukoshan Fm (early-late Pleistocene; Fig. 1b)^22,23,24^. The overall shallowing-upward trend preserved in the Chinshui-Cholan-Toukoshan succession reflects the westward migration of the Taiwan Orogeny^20,35^.

## Results

### Magneto-biostratigraphy

**Figure 3:**
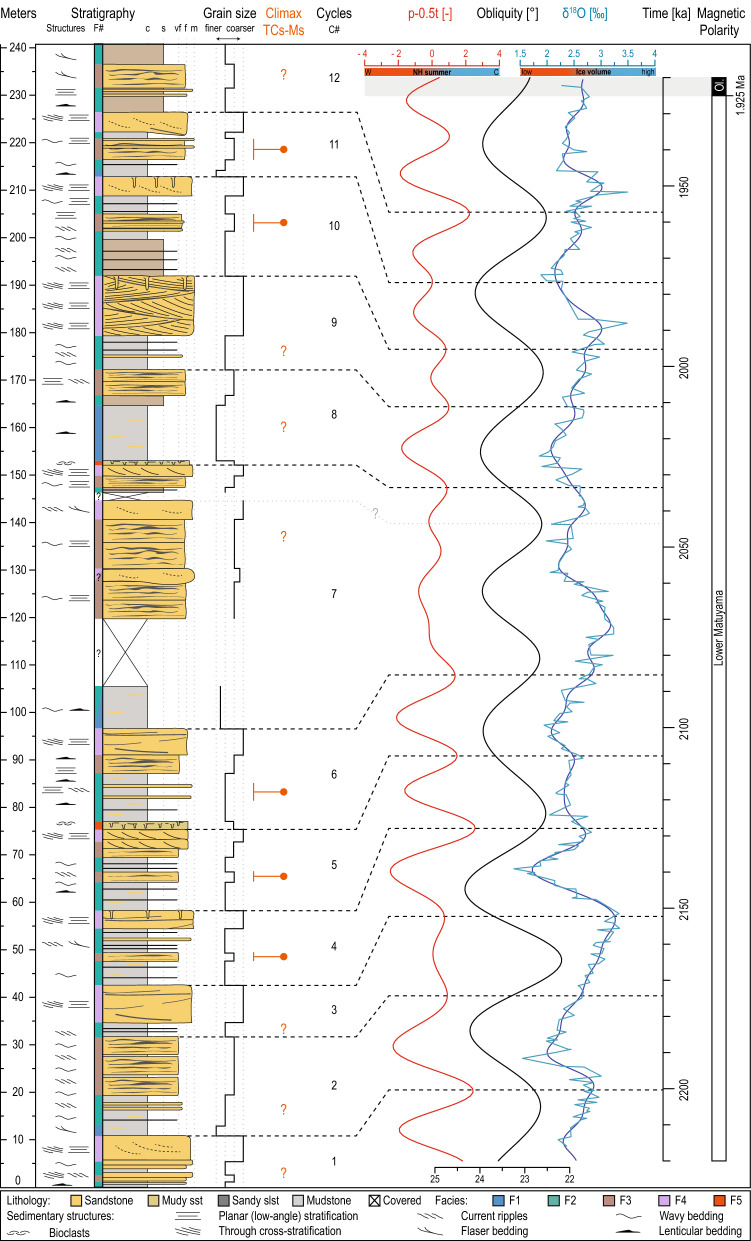
Stratigraphy and chronology of the lower Cholan Fm along the Houlong River. The stratigraphic data (facies, lithology, grain size) are compared and correlated to: (1) a mix-standardized precession minus 0.5 times standardized obliquity (p-0.5t)^39^ as orbital reference based on the Laskar, et al.^7^ solution (red curve), (2) pure obliquity curve (black curve)^7^, and (3) a δ^18^O record from benthic foraminifera (light blue curve)^40^ smoothed (dark blue curve) using a Taner^41^ low-pass filter with a cutoff frequency of 0.1 and a roll-off rate of 10^10 using the ‘astrochron’ R package^42,43^. Climax periods of tropical cyclone and monsoon are pointed out by orange dots or orange “?”. W: warmer, C: colder. The illustration was made using Adobe Illustrator CS6 (https://www.adobe.com/).

Paleomagnetic data resolve a normal-reversed-normal-reversed magnetic polarity sequence upwards through the Cholan Fm (Fig. 1c; see SI). The lower normal polarity zone recorded in sites CL01 to CL09 (Fig. 1c) correlates to the upper Gauss chron (3.032–2.595 Ma; Fig. 1c) because these strata contain the calcareous nannofossil *Discoaster surculus*^29,30^. The upper normal polarity zone recorded in sites CL37 to CL48 (Fig. 1c) is below the first appearance of medium *Gephyrocapsa* (> 4 μm in size) and consequently is correlated to the Olduvai subchron (1.925–1.780 Ma)^29,30^. Based on these constraints, the lower reversed polarity zone, which contains the studied stratigraphic interval (CL15 to CL36), occurs within the lower Matuyama chron close to the lower limit of the Olduvai (1.925 Ma) subchron. These strata have an estimated sedimentation rate of 96 ± 36 cm.kyr^−1^ (Figs. 1c, 3).

### Depositional environments

Repeated coarsening upward cycles are expressed in the stratigraphy of the lower Cholan Fm along the Houlong River (Figs. 2, 3). Five sedimentary facies constitute the sedimentary succession (Fig. 3, see SI), including: massive to laminated mudstone (F1); mudstone-dominated heterolithics (F2); sandstone-dominated heterolithics (F3); cross-bedded sandstone (F4); and bioturbated muddy sandstone (F5). F1 and F2 are mudstone-dominated facies, and are interpreted as the product of mud deposited in offshore environments. Thin sandstone laminae in F1 and top-down burrowed, sharp-based sandstone beds in F2 are interpreted as event deposits (e.g., tropical cyclone (TC) beds). The sandstone-dominated facies, F3 and F4, are interpreted as being deposited in proximal offshore to nearshore environments. Sedimentary structures in F3 and F4 indicate that the currents that impacted sedimentation were mainly north- and south-directed, and potentially record bidirectional tidal flow. Tidal influence is also suggested by the occurrence of mudstone drapes on the foresets of trough cross-stratification. Bioturbated muddy sandstone of F5 is the least common facies in the stratigraphy and is interpreted as recording bioturbated seafloor deposits formed under reduced sedimentation rates and well-oxygenated conditions.

In addition to primary sedimentary structures, the ichnology of the facies varies significantly and reveal details about the paleoenvironment. Facies 5 is completely bioturbated (Bioturbation Index; BI 6), while F1 to F4 show low to moderate bioturbation intensities (BI 0–4). The reduced bioturbation in F1 to F4 is attributed to high sedimentation rates and fresh-water influx in the environment during accumulation of these facies, and the erosion of strata that was colonized by infauna during periods of low sedimentation. Together, F1 to F5 are interpreted as being deposited in offshore to nearshore environments^37^ subject to tidal action and under the influence of rivers that increased discharge during TCs^26,44^.

The different facies that define depositional cycles depict an overall shallowing-upward pattern with a lower interval displaying offshore environments (F1 and/or F2) passing upwards into an interval that preserves deposition in nearshore environments (F3 and/or F4 rarely F5; Figs. 2, 3). Of note, several cycles show an increase of sand content halfway up the shallowing-upward cycle but within facies identified as offshore deposits. These sand beds are expressed as either individual beds or as heterolithic intervals (orange dots; Figs. 2, 3), and are interpreted as event beds (probably TC beds). Twelve depositional cycles (C1–C12; Figs. 2, 3) are defined in the vertical succession. The lowermost (C1) and uppermost (C12) cycles are partially covered by vegetation, and hence, are incomplete. The remaining 10 cycles (C2–C11), excluding C7, have a relatively consistent thickness of 18.7 ± 4.6 m (± 1 σ). Cycle C7 is 55.3 m thick.

### Correlation to climate curves

In order to pinpoint the triggering mechanisms responsible for sea-level changes and their timing, we compare our stratigraphic data against (1) a δ^18^O isotopic record from benthic foraminifera considered as an ice-volume proxy^40^, (2) a pure obliquity curve^7^, and (3) a mix of standardized precession minus 0.5 times standardized obliquity/tilt (p-0.5t)^39^ as orbital reference based on the Laskar, et al.^7^ solution (Fig. 3). The p-0.5t curve mimics the insolation received at ~ 65° latitude in the Northern Hemisphere but does not make a link to any specific latitude. Commonly, depositional cycles are correlated to the δ^18^O curve where low δ^18^O values suggest a low ice-volume and high sea level and vice versa. As stated previously, the studied interval is within the lower Matuyama chron and close to the lower limit of the Olduvai subchron (1.925 Ma). Consequently, the δ^18^O, obliquity, and p-0.5t curves are anchored at 1.925 Ma, where the Matuyama-Olduvai contact is defined in the studied section (Fig. 3).

A comparison of the three curves (δ^18^O, obliquity, and p-0.5t, Fig. 3) shows that the δ^18^O record displays an obliquity signal but also has a precession component underlined by the Taner filter. The obliquity data correlates with the δ^18^O data with an offset, where the offset represents a time lag due to the inertia of the ice sheets^45,46^. There is a good correlation between the p-0.5t and δ^18^O curves because both datasets have both obliquity and precession components; however, in the p-0.5t curve, precession dominates.

To estimate the duration of cycles we use the sedimentation rate calculated by Chen, et al.^19^ whom developed a magneto-biostratigraphic framework for the same section and we use the updated age of paleomagnetic polarity boundaries^29^. The sedimentation rate from Chen, et al.^19^, ~ 110 cm kyr^−1^, is more reliable than our rate (i.e., 96 ± 36 cm kyr^−1^) because they included samples taken near the polarity boundaries. The calculated sedimentation rate from Chen, et al.^19^ gives an estimated duration of the studied interval of ~ 220 kyrs, which translates into an average duration of 17 ± 4 kyrs per sedimentary cycle (excluding C7). The duration of the cycles approximates the duration of precession cycles, although, variations in the sedimentation rate could have occurred during the lower Matuyama chron. Consequently, we studied the reference data from the curves in a flexible range of sedimentation rates and suggest a correlation in Fig. 3. Depositional cycles of the lower Cholan Fm show the greatest similarity to the p-0.5t curve rather than the δ^18^O curve. As such, we correlate depositional cycles to the p-0.5t curve while anticipating a time lag due to the inertia of the ice sheet.

We based our correlation of stratigraphy to orbital forcing on the following assumption. A minimum value of p-0.5t corresponds to warmer summers in the Northern Hemisphere. Warmer summers induced partial melting of Northern Hemisphere ice sheets and this caused eustatic sea-level rise. Based on this, we correlate minima in the p-0.5t curve to offshore environments (higher sea level and mudstone-dominated intervals), and p-0.5t maxima to nearshore environments (lower sea level and sandstone-dominated intervals).

The stratigraphy of the lower Cholan Fm, with the exception of cycle C7, consists of shallowing-upward cycles that are 18.7 ± 4.6 m-thick (Fig. 3). C7 is the thickest cycle, and all cycles display a relatively consistent vertical arrangement of facies. In the p-0.5t curve, the only interval that differs from the rest of the curve occurs between 2.086 and 2.033 Ma, and during this time, the p-0.5t curve shows reduced amplitude fluctuations. We tie the reduced amplitude fluctuations to the C7 cycle because it is the only depositional cycle that shows reduced environmental variation over a relatively protracted period. Correlating intervals of reduced paleoenvironmental variability to minima in eccentricity and precession amplitudes is common practice in astrochronological studies^47^. Consequently, we use C7 to anchor the stratigraphy to the astrochronologically-tuned δ^18^O, obliquity, and p-0.5t curves (Fig. 3). We describe and correlate cycles below and above C7. Using both the sedimentation rate (and corresponding cycle frequency based on sedimentation rate) and variations in the various curves allows the least ambiguous assignment of sedimentary cycles to orbital variations^47,48^.

Below C7, C4–C6 comprise three coarsening upward cycles. The three sandstone-dominated intervals at the tops of C4–C6 correlate to three maxima in the p-0.5t curve, and their strong amplitudes at 2.128, 2.108, and 2.086 Ma. These three maxima correlate to nearshore facies at 58.2 m, 75.5 m, and 96.7 m, respectively. Below C4, cycles C2 and C3 also show coarsening-upward trends, although C2 has a finer grain-size; this is interpreted as recording deposition close to the nearshore-offshore limit. The top of nearshore facies at 42.5 m and 32 m correlate to weaker maxima in the p-0.5t curve at 2.174 and 2.153 Ma. Consequently, the upper part of cycle C1 correlates to the p-0.5t maximum at 2.201 Ma. Above cycle C7, the p-0.5t curve shows one marked minimum, and four maxima. The p-0.5t minimum at 2.022 Ma correlates to the thick offshore interval of C8. The nearshore intervals of C8–C11 at 172.2 m, 192 m, 212.8 m and 226.4 m correlate to the four maxima at 2.012, 1.996, 1.977 and 1.958 Ma, respectively.

In summary, the combination of (1) the anchor at the lower limit of the Olduvai normal polarity, (2) the sedimentation rates suggesting precession duration of the depositional cycles, (3) the low environmental variations of C7 tied to the reduced amplitude fluctuations of the p-0.5t curve, (4) a quasi-cyclic expression of sedimentary cycles matching to p-0.5t cycles, and (5) inferred sea level amplitude matching the p-0.5t curve all demonstrate the validity of our astrochonological framework and highlight that the stratigraphy of the lower Cholan Fm is paced by insolation received in the Northern Hemisphere, which is dominated by precession.

## Discussion

### Insolation-paced sea-level changes

Depositional cycles in the lower Cholan Fm record changes in depositional environments through time, and these changes are interpreted to reflect quasi-cyclic precession-dominated sea level fluctuations in the paleo-Taiwan Strait (Figs. 2, 3). Milankovitch^2^ proposed that summer insolation, driven by a combination of obliquity and precession, led to ice-volume changes, suggesting that both obliquity and precession signals should be expressed in glacial records. However, during the late Pliocene–early Pleistocene, the growth and decay of ice sheets is commonly attributed to changes in obliquity that translates into ~ 41 kyr-frequency sea-level fluctuations^4,5,15,49,50,51,52,53,54^. The interpretation of obliquity-forced glacial cycles is widely accepted despite weak precession signals preserved in glacial records^4,40,50^.

Marine archives, and more specifically ice-volume and deep-water temperature proxies are dominated by obliquity signals during the early Pleistocene because of the in-phase effect of obliquity-related insolation *versus* the opposite-phased influence of precession. The opposite-phased influence may have canceled out the summer insolation signal received by the northern and southern hemispheres^5,51,52,55^. Nevertheless, recent findings showcase that precession played a more important role than previously thought in sea-level cycles during the Pliocene^16^ and glacial cycles during the early Pleistocene^56^. Indeed, δ^18^O records from the North Atlantic are almost in-phase with the Northern Hemisphere summer insolation during the early Pleistocene^56^. Liautaud, et al.^56^ proposed two possible scenarios in which precession acted on glacial cycles during the early Pleistocene: (1) Northern Hemisphere summer insolation mainly paced glaciations, and/or (2) precession-driven ice-volume changes shifted from affecting mainly Southern Hemisphere ice sheets in the late Pliocene to Northern Hemisphere ones in the early Pleistocene. These hypotheses imply that during the early Pleistocene, the Antarctic ice sheets were relatively stable, and global cooling favored the southward expansion of Northern Hemisphere ice sheets. The latter suggests that Northern Hemisphere ice sheets were more sensitive to ablation induced by precession-paced summer insolation, and this had a more substantial impact at lower latitudes^2,5,52,56^. Together, these studies propose that precession had a significant influence on glacial cycles during the early Pleistocene by driving change in the insolation received by the Northern Hemisphere.

Our results show that the depositional cycles in the lower Cholan Fm are primarily driven by changes in summer insolation in the Northern Hemisphere, and this is precession-dominated with an obliquity component (Fig. 3). Specifically, depositional cycles in the lower Cholan Fm preserve evidence of insolation-paced sea-level changes during the early Pleistocene and in Southeast Asia. These sea-level fluctuations shifted facies belts and potentially had amplitudes (13 ± 5 m) that were similar to those identified in late Pliocene strata (3.3 to 2.5 Ma) in New Zealand, the latter of which were linked to precession-paced sea-level cycles^16^. However, since summer insolation has an obliquity component, which is reflected in the amplitude variations in the p-0.5t curve, it is possible that the low amplitude sea-level fluctuations induced by precession were modulated by obliquity such that sea-level amplitude variations were significantly higher (~ 50 m)^53,54^ as is generally expected during the early Pleistocene.

Sea-level reconstructions are mostly derived from the δ^18^O isotopic records of benthic foraminifera^53,57,58^, which are considered to be temperature proxies, and enable calculations of changes in ice volume with changing temperature. However, Rohling, et al.^54^ inferred that ice-sheet growth might not be expressed directly in the δ^18^O isotope record, and ice-sheets potentially exceeded the volumes estimated based on paleo-temperature records. This would reinforce that precession cycles are not well expressed in δ^18^O records, but still impacted glacial cycles^56^. In addition, small sea level cycles are preferentially recorded in the shallow-marine stratigraphy where sea-level variations have a major impact on the position of facies belts and stratigraphic architecture^59,60,61^. Shallow-marine records are commonly disregarded as continuous climate archives, which partly explains why precession-dominated sea-level fluctuations remain poorly identified in rock-record paleoclimate studies globally.

### Sediment flux forced by Northern Hemisphere summer insolation

Beyond the role of summer insolation in driving glacial cycles and their associated quasi-periodic sea-level fluctuations, precession also has a strong effect on hydrological cycles at low latitudes^9,10,11^. In the lower Cholan Fm, depositional cycles commonly show an increase in sand content in the middle of cycles and in offshore environments (orange dots; Figs. 2, 3) suggesting that a secondary process impacted deposition. Presently, precipitation in Taiwan is evenly distributed between TCs (52.5%) and monsoons (47.5%)^62^. However, more than 75% of sediment delivered to the Taiwan Strait occurs during TCs^63,64,65^ and the volume of sediment exported during TCs varies significantly depending on whether the TC interacts with monsoon flows or not^66^. On orbital timescales, the global monsoon system is controlled dominantly by precession resulting in higher monsoon intensity at low latitudes, and this alternates between hemispheres every ~ 10 kyrs^11,67,68,69,70^.

**Figure 4:**
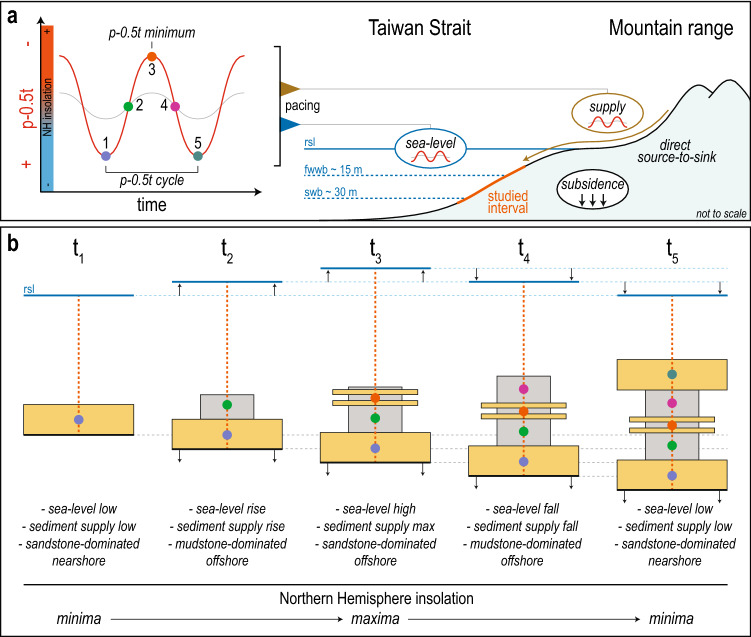
(**a**) Conceptual model of depositional cyclicity recorded in the lower Cholan Formation. The three main actors that influence deposition are sea level, sediment supply, and subsidence (red curve: large amplitude p-0.5t cycle; gray line: low amplitude p-0.5t cycle). (**b**) While subsidence is considered continuous, sea level and sediment supply fluctuated following the Northern Hemisphere (NH) insolation (p-0.5t curve), which is dominated by precession (Fig. 3). When the NH insolation increases (1 to 2), sea level rises due to the partial melting of NH ice sheets, and sediment supply increases through the coupled action of tropical cyclones and monsoons. The NH insolation maximum (3) relates to the highest sea level and the climax of the coupled tropical cyclones and monsoons, which are preserved as an increase of sand exported into offshore environments. Next, the NH insolation decreases (4), and sea level falls and decreases sediment supply (4). The lowest points of both sea level and sediment supply are reached when summer insolation of the NH is minimum. rsl: relative sea level; fwwb: fairweather wave base; swb: storm wave base. The illustration was made using Adobe Illustrator CS6 (https://www.adobe.com/).

Paleotemperature proxies indicate that precession had an impact on global temperature (Fig. 3)^4,40,56^, and increasing temperature had two major impacts on TCs: (1) both their frequency and strength increased^71,72,73,74^, and (2) their translation speeds decreased leading to more precipitation on land^75,76^. Consequently, temperature variations via precession-paced insolation during the early Pleistocene impacted both TC translation speeds and rainfall, which, in turn, impacted sediment erosion on land and export to the Taiwan Strait. Precession, via variations in summer insolation, primarily forced sediment supply to the Taiwan Strait thought the coupled action of TCs and monsoons (TCs-Ms). TCs-Ms promote erosion on land and increases the export of sediment to the paleo-Taiwan Strait (Figs. 2, 3, 4). In the Cholan Fm, the climax of TCs-Ms correlates to sandier intervals in offshore facies (orange dots; Figs. 2, 3, 4) recording an increase in sand exported into offshore environments during the early Pleistocene. Such increases in sediment supply relate to periods of warmer Northern Hemisphere summers (p-0.5t minima) and correspondingly high eustatic sea levels.

Taiwan is the largest sediment contributor to the world’s oceans relative to its size^77^ with 174 to 384 Mt of sediment delivered annually to the surrounding seas^32^. Taiwan-sourced sediment dominates deposition in the Taiwan Strait^34,77,78,79^ and this was probably similar during the early Pleistocene^27,37^. Regular earthquakes trigger landslides into river catchments and these sediments are mobilized and exported to the ocean during subsequent TCs^32,33,63,80^. Intense precipitation, erosion, and sediment transport during TCs-Ms significantly increase the amount of sediment exported to the seas surrounding Taiwan, and TCs-Ms are affected by climate oscillations. The short river systems on the island promote a direct source-to-sink relationship between erosion and deposition, such that climate perturbations are “directly” manifested in the sedimentary record of the paleo-Taiwan Strait. The high sediment supply and virtual absence of a transfer zone (i.e., extensive flood plains), that could potentially buffer climatic signals^81^, increase the amount of sediment exported. Moreover, the short transfer zone increases the preservation of the climax of the TCs-Ms, which correlates to the maximum summer insolation received in the Northern Hemisphere (Fig. 4).

### Conceptual model for insolation-paced sea level and sediment supply

During the early Pleistocene, the WFB was affected by substantial subsidence, and as demonstrated herein, both sea level and sediment flux were paced by summer insolation received in the Northern Hemisphere (Fig. 4). The conceptual model we put forward explains how the stratigraphy of the lower Cholan Fm accumulated following a p-0.5t cycle during which subsidence was considered constant (1 to 5, Fig. 4). Insolation drove changes in ice volume; although, there is a time lag in the response of ice-sheets to insolation suggesting that the p-0.5t curve (precession) was not the dominant driver of ice-volume change. In the theoretical model, deposition starts at a sea-level low (Northern Hemisphere insolation minimum), at which time, nearshore environments were sand-dominated (1, Fig. 4). As insolation increased (1 to 2, Fig. 4), sea level started to rise due to the partial decrease in ice volume in the Northern Hemisphere, and this shifted facies belt landward. At the same time, sediment supply increased due to the strengthened action of TCs-Ms that increased the export of mud to offshore environments. During the insolation maximum, which coincided with the p-0.5t minimum, sea level reached its highest stand and the climax of TCs-Ms is manifested through an increase of sand export to offshore environments (3, Fig. 4). Subsequently, Northern Hemisphere insolation decreased and induced a fall in sea level; this shifted facies belts basinward and reduced sediment supply (4, Fig. 4) before reaching the lowest point for both sea level and sediment supply (5, Fig. 4).

The conceptual model depicts what happens when p-0.5t has strong amplitudes as it mostly did during deposition of the lower Cholan Fm. However, the p-0.5t amplitude is weaker in some intervals (gray curve; Fig. 4a, see also C7 in Fig. 3), suggesting variations exist in sea-level values and sediment supply cycles (C7, Fig. 3).

## Conclusion

This study provides the first stratigraphic evidence of insolation-paced sea-level changes in Southeast Asia that is dominated by precession; this is in contrast to the obliquity-dominated records preserved in marine archives during the early Pleistocene globally. Our findings are supported by a high degree of similarity between shallowing-upward cycles (offshore to nearshore) of the lower Pleistocene Cholan Formation (Taiwan) and the p-0.5t curve that represents the precession-dominated insolation received by the Northern Hemisphere. The studied interval is temporally constrained by magneto-biostratigraphy to the lower part of the Matuyama chron, and that correlation coupled with astronomical tuning indicates that the studied interval extends from 2.21 to 1.96 Ma. During deposition, insolation maxima received in the Northern Hemisphere promoted partial ablation of ice sheets inducing variations in eustatic sea level (potentially 13 ± 5 m although higher values might occur), which varied the position of facies belts. The direct source-to-sink system defining sediment routing on Taiwan allows for tracking sediment supply changes in response to climate oscillations in the shallow-marine realm. The insolation received in the Northern Hemisphere affected both tropical cyclones and monsoons, and these processes varied sediment supply. The climax of tropical cyclones and monsoons coincided with insolation maxima, and are expressed as an increase of sand recorded in offshore environments.

The tropical climate (i.e., tropical cyclones, monsoons) and fast-growing Taiwan Orogeny facilitated the completeness of the stratigraphic record on an orbital time scale. Our study underpins that shallow-marine strata from basins experiencing conditions of high accommodation-space generation and hinterland weathering are outstanding climate archives, from which concrete narratives of Earth's past history can be extracted.

## Methods

### Magnetostratigraphy and biostratigraphy

The chronostratigraphic framework of the Cholan Fm developed herein is based on 76 sites (CL01 to CL76) where paleomagnetic cores were collected and analyzed for their remanent directions (Fig. 1C). Sites were located according to Global Positioning System (GPS) readings by using a portable Garmin GPSMAP 60CSx. Intervals between sites were then determined based on GPS data and structural information of the strata (dip and strike). For each paleomagnetic site, 2–3 cores (25 mm in diameter) were drilled from mudstone after removing the weathered surface, and cores were oriented (azimuth and dip) with a magnetic compass and an orientation tool. Additional samples were taken at some paleomagnetic sites for calcareous nannofossil analysis. The 241 m-thick section (Fig. 3) start 14 m below the sampling site (CL15; Fig. 1C) because the lowermost part of the outcrop is sandy and was not accessible with the drilling machine. The paleomagnetic and biostratigraphic analyses were made at the Institute of Earth Sciences, Academia Sinica, Taipei, Taiwan. Information about the sampling sites, remanent directions for selected samples during thermal demagnetization analysis, data sources, and methodology used to develop the magneto-biostratigraphic framework are presented in Supplementary Information 1.

### Sedimentology

The 241 m-thick stratigraphic succession described herein belongs to the lower Cholan Fm (Figs. 2, 3). These strata were logged at a decimeter scale, and observations were made regarding bed geometries, bounding contacts, grain size, sedimentary structures, ichnology, and body fossils. Outcrop images were acquired using a DJI Mavic 2 Pro drone. Illustrations and descriptions of the sedimentary facies are provided in Supplementary Information 2.

### Astrochonology and correlation

For comparison to our lithological log, we use a δ^18^O record from benthic foraminifera, which mainly represents global ice volume and deep-sea temperatures. Due to its independent age scale by orbital tuning of physical property data, we use the equatorial Atlantic dataset of Wilkens, et al.^40^ which is similar to the LR04 stack^4^. The data was smoothed using a Taner^41^ low-pass filter with a cutoff frequency of 0.1 and a roll-off rate of 10^10 using the ‘astrochron’ R package^42,43^. Further, we use a mix-standardized precession minus 0.5 times standardized obliquity^39^ as orbital reference based on the^7^ solution. The used R code is in the Supplementary Information 3.

## Supplementary Information

Supplementary Information.

## Data Availability

The dataset generated and analyzed in this study are included in this published article (and its Supplementary Information).
