# Supplementary material for: Insolation-paced sea level and sediment flux during the early Pleistocene in Southeast Asia
Source: Sci Rep. 2021 Aug 18;11:16707. doi: 10.1038/s41598-021-96372-x (PMC8373940; doi:10.1038/s41598-021-96372-x)
Supplement: Supplementary file 1 — Supplementary Information. [file 41598_2021_96372_MOESM1_ESM.docx]

Supplementary Information

**Insolation-paced sea level and sediment flux during the early Pleistocene in Southeast Asia**

Romain Vaucher^1*^, Shahin E. Dashtgard^1^, Chorng-Shern Horng^2^, Christian Zeeden^3^, Antoine Dillinger^1^, Yu-Yen Pan^4,5^, Romy A. Setiaji^5^, Wen-Rong Chi^6,7^, Ludvig Löwemark^5^

*^1^Applied Research in Ichnology and Sedimentology (ARISE) Group, Department of Earth Sciences,*

*Simon Fraser University, Burnaby, Canada*

*^2^Institute of Earth Sciences, Academia Sinica, Taipei, Taiwan*

*^3^LIAG – Leibniz Institute for Applied Geophysics, Geozentrum Hannover, Hannover, Germany*

*^4^Centre for Natural Hazards Research, Department of Earth Sciences, Simon Fraser University, Burnaby, Canada*

*^5^Department of Geosciences, National Taiwan University, Taipei, Taiwan*

*^6^Department of Earth Sciences, National Central University, Taoyuan, Taiwan*

*^7^Department of Earth Sciences, National Cheng-Kung University, Taïnan, Taiwan*

***Corresponding author: [romain.vaucher88@gmail.com](mailto:romain.vaucher88@gmail.com)

**This PDF file includes:**

Supplementary text

Figures S1 to S5

Tables S1 to S2

References

Supplementary Information 1: Magneto-biostratigraphy p. 2–6

Supplementary Information 2: Sedimentary Facies p. 7–9

Supplementary Information 3: R code p. 9

References p. 9–10

**Supplementary Information 1: Magnetobiostratigraphy**

**Supplementary text**

The magnetobiostratigraphic framework for the Cholan Formation was previously established ^1^, however the identified Olduvai and the upper Gauss normal polarity zones cannot be located precisely on the map because there were no GPS data of the sampling sites at that time. In this study we re-sampled the Cholan Formation to build a new magnetobiostratigraphy framework to accurately justify the relationship of the studied interval with the geomagnetic polarity records. The hereafter paleomagnetic and biostratigraphic analyses were made at the Institute of Earth Sciences, Academia Sinica, Taipei, Taiwan.

***Paleomagnetic analysis***

Paleomagnetic cores (25 mm in diameter) were oriented and collected from 76 sites (CL01-CL76) with GPS data for most of sites (Fig. S1 and Table S1). Non-oriented samples were also taken from the sites for studying magnetic mineralogy. The space between sampling sites is mostly less than 30 m, but can be greater when the outcrop is covered (see Table S1; Fig. S1). Samples (22 mm in length) were cut from oriented cores in the laboratory and were then subjected to paleomagnetic analysis. The natural remanent magnetization (NRM) of samples was measured at room temperature (i.e., 25°C) with a 2-G Enterprises superconducting rock magnetometer. A Magnetic Measurements Ltd. thermal demagnetizer with a low-field cooling chamber was used for stepwise thermal demagnetization of the samples from room temperature to 600°C (i.e., 25, 120, 160, 200, 240, 280, 300, 320, 340, 360, 380, 400, 420, 440, 460, 480, 520, 560, and 600°C). To monitor possible changes in magnetic minerals and remanent magnetizations during heating, low-field magnetic susceptibility was measured on samples after each heating step with a Bartington Instruments MS2B magnetic susceptibility system. To characterize magnetic mineralogy in samples and to interpret the behavior of remanent magnetization during thermal demagnetization, magnetic mineral separation from non-oriented samples using a rare earth magnet housed in a plastic sheath was done. Then X-ray diffraction (XRD) analysis for mineral identification was carried out on magnetic extracts using a Rikagu Miniflex table top unit (X-ray scans were run from 4 to 80^o^ (2θ) under Cu-Kα radiation). XRD spectra for a representative sample is shown in Figure S2. After stepwise thermal demagnetization was completed for each sample, a characteristic remanent magnetization (ChRM) direction (declination and inclination) and its magnetic polarity either normal or reversed were identified using data from at least 4 demagnetization steps with principal component analysis ^2^ after removal of a viscous secondary magnetization. A detailed data-processing is demonstrated in Figure S3 and nine representative thermal demagnetization diagrams of samples from studied sites were selected and are shown in Figure S4 and a new magnetostratigraphy based on ChRMs is constructed (Fig. 1C).

***Calcareous nannofossil analysis***

Smear slides were prepared for calcareous nannofossil identification from small chips of bulk sediments and were examined under an optical microscope with 1000*×* magnification. Among the nannofossil species, *Discoaster* *surculus* and the medium *Gephyrocapsa* spp. (≥ 4 μm in size) are of our interest because the last appearance and the first appearance ages for the former and latter species are 2.53 Ma and 1.71 Ma, respectively, which are slightly younger than the upper Gauss and the Olduvai normal polarity subchrons ^3, 4^ (Fig. 1C). Thus, the two nannofossil species provide time constraints on the identified paleomagnetic polarity zones in the Cholan Formation. It is noted that the abundance of nannofossils is generally few or even barren because sediments of the Cholan Formation were deposited in shallow-marine environments.


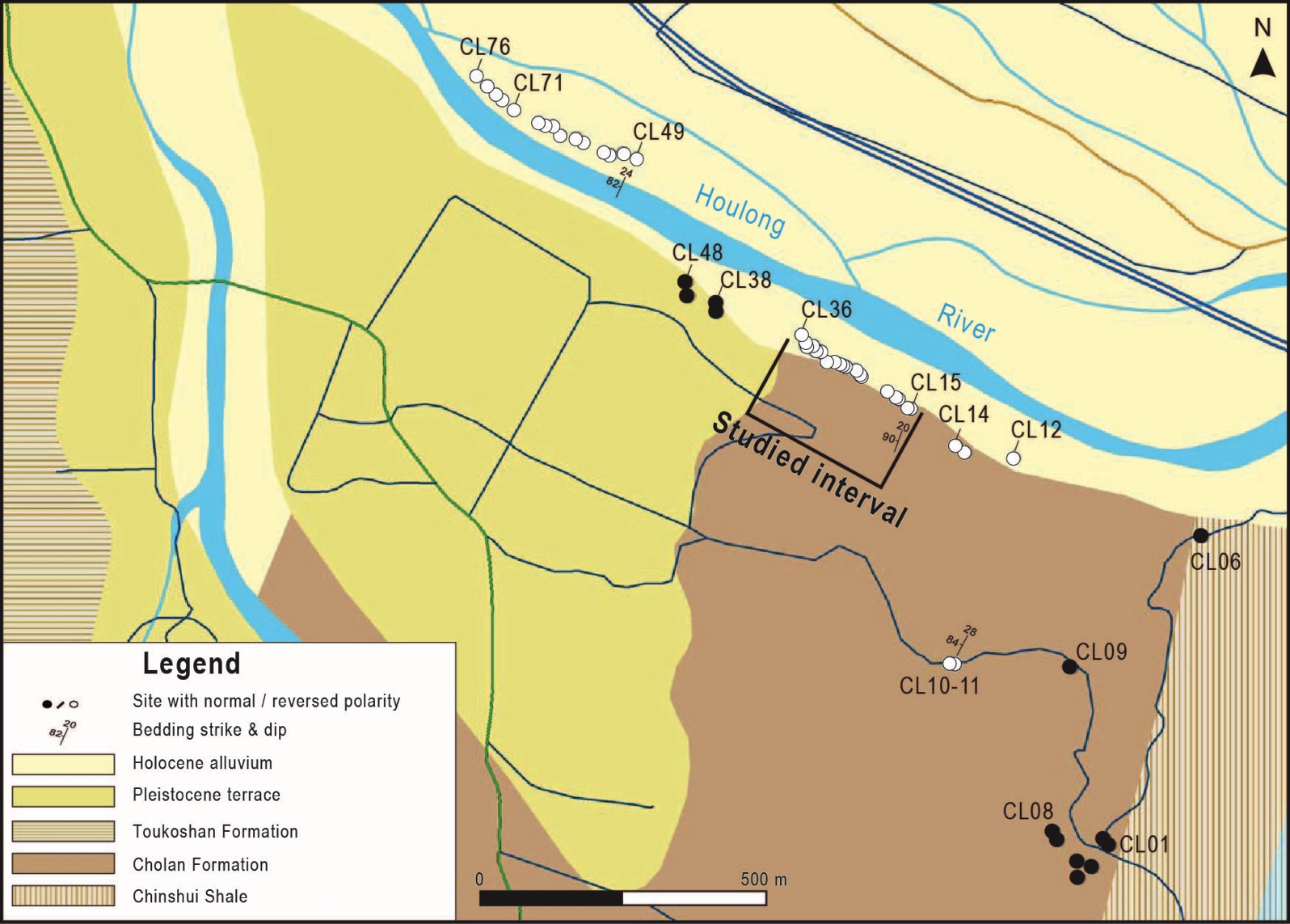


**Figure S1:** Geological map of the studied area showing the 76 sites of samples used to develop the magnetobiostratigraphic framework. The base and the top of the Cholan Formation are contacted conformably with the Chinshui Shale and the Toukoshan Formation, respectively. Bedding planes (strikes: 20-30^o^ E and dips: 80-90^o^ W) and magnetic polarity records of the Cholan Formation are also shown. Paleomagnetic and GPS data are given in Table S1. The map is modified from TungShih geological map published by Central Geological Survey, MOEA ^5^.

******

**Figure S2**: X-ray diffraction spectra in black for a magnetic extract separated from a sample at Site CL13, indicating that the magnetic mineral in the Cholan Formation is mainly composed of titanomagnetite. Since titanomagnetite is a complete solid solution series (Fe_3-x_Ti_x_ O_4_, 0≦x≦1), its Curie temperature may ranges from 176^o^C (i.e., Fe_2.4_Ti_0.6_O4) to 578^o^C (i.e., Fe_3_O_4_) depending on the substitution amount of Ti for Fe ^6^, which therefore may also affect the thermal stability of remanent magnetization (see Figure S3).


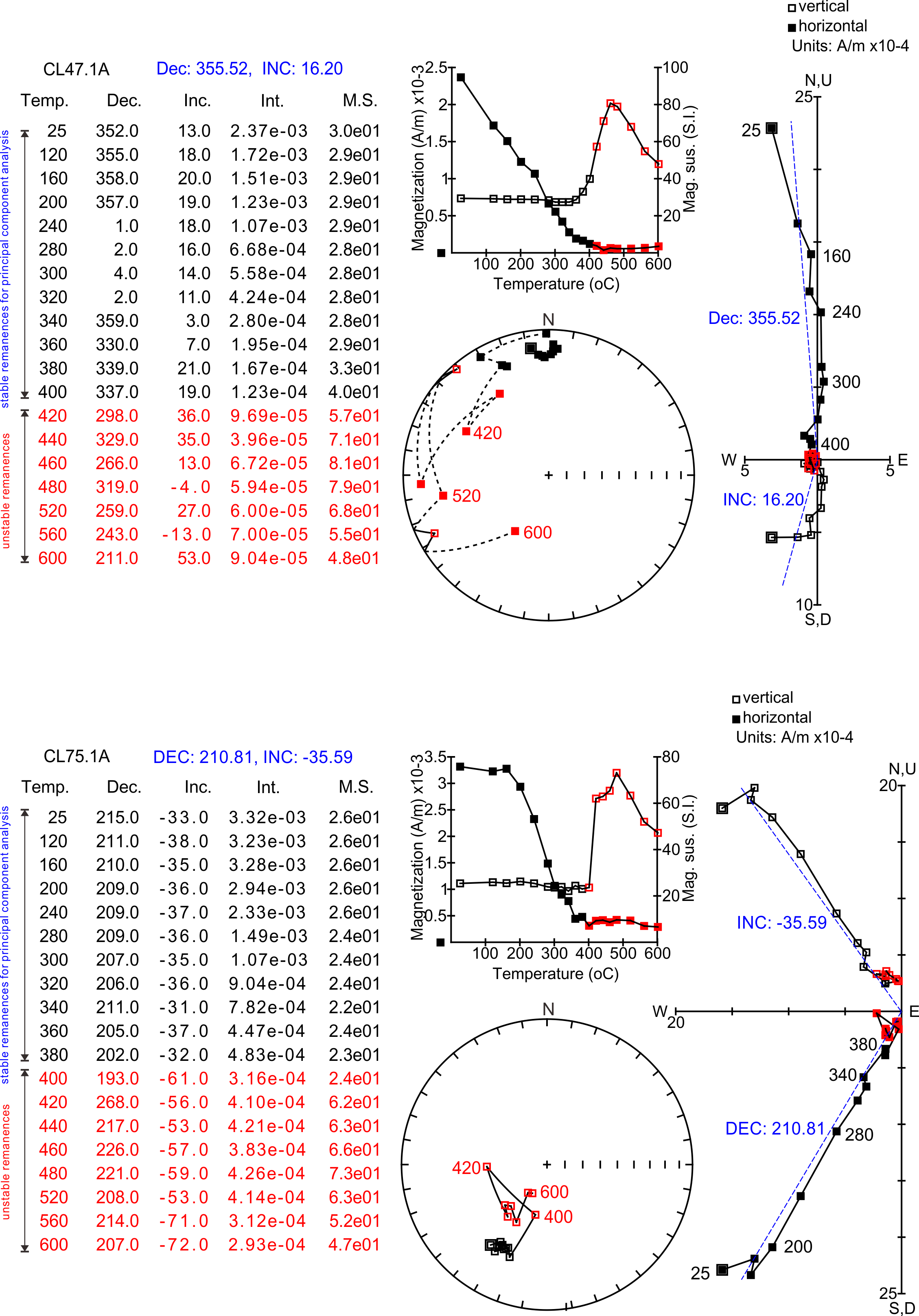


**Figure S3**: Complete stepwise thermal demagnetization results (temperature step, declination, inclination, intensity of remanent magnetization, and magnetic susceptibility) and data plots (orthogonal and stereographic) for two representative samples. Stable magnetic remanences at lower temperature steps were used for principal component analysis and their mean directions toward the origin point are shown with blue dashed lines. Unstable remanences at higher temperature steps shown in red were not used.

**Figure S4:** Thermal demagnetization diagrams for 9 selected paleomagnetic samples from the Cholan Formation. Solid squares (◼): horizontal projections (declinations); open squares (☐): vertical projections (inclinations). Values next to the solid squares are temperatures (°C). Intensity of NRM at 25^o^C (Int_25_, 10^-4^ A/m) of each sample is indicated. The declinations and inclinations displayed in each plot are stable remanent directions (blue dashed lines) of each sample after principal component analysis. Paleomagnetic results yielded a reliable normal (a-b)-reversed (c-d)-normal (e-f)-reversed (g-i) magnetic polarity stratigraphy from the base to the top of the Cholan Formation (see Fig. 1C).

**Table S1:** Details of the magnetobiostratigraphic dataset for the Cholan Formation of the Houlong River section. Gray: normal polarity; white: reversed polarity

| **Site** | **Sample** | **Latitude (N°)** | **Longitude (E°)** | **Level (m)** | **Declination_ChRM** | **Inclination_ChRM** | **Nannofossils** |
| --- | --- | --- | --- | --- | --- | --- | --- |
| CL01 | YF15.2A | 24,456509 | 120,824649 | 1538,0 | -2,0 | 46,2 |  |
| CL02 | YF16.1A | 24,456608 | 120,82456 | 1526,4 | -6,2 | 18,2 |  |
| CL03 | YF17.1A | 24,456157 | 120,824353 | 1524,2 | -6,0 | 24,2 |  |
| CL04 | YF1.25B | 24,455985 | 120,824107 | 1508,2 | 16,4 | 42,2 |  |
| CL05 | YF1.20A | 24,456247 | 120,824106 | 1495,8 | -5,9 | 27,9 |  |
| CL06 | YF2.1A | 24,461441 | 120,82626 | 1492,8 | 2,5 | 21,1 | *Discoaster surculus* |
| CL07 | YF20.1A | 24,456589 | 120,823751 | 1449,8 | -1,0 | 19,6 |  |
| CL08 | YF21.1A | 24,456725 | 120,823672 | 1437,0 | -1,4 | 23,4 | *Discoaster surculus* |
| CL09 | YF24.1A | 24,459343 | 120,823984 | 1363,9 | 18,7 | -2,6 |  |
| CL10 | HLC090.3A | 24,459395 | 120,821942 | 1170,7 | 183,1 | -33,0 |  |
| CL11 | HLC089.4A | 24,459413 | 120,821863 | 1158,3 | 196,7 | -60,2 |  |
| CL12 | HLC047.1A | 24,462692 | 120,822973 | 1131,7 | 220,6 | -46,1 |  |
| CL13 | HLC046.1A | 24,462772 | 120,822115 | 1048,8 | 197,0 | -46,0 |  |
| CL14 | HLC045.1A | 24,46288 | 120,821957 | 1027,8 | 178,4 | -39,4 |  |
| CL15 | HLC044.1A | 24,463466 | 120,821177 | 928,9 | 193,9 | -32,0 |  |
| CL16 | HLC043.1A | 24,463484 | 120,821108 | 922,1 | 172,0 | -24,6 |  |
| CL17 | HLC042.1A | 24,46362 | 120,820959 | 902,5 | 190,8 | -32,0 |  |
| CL18 | HLC041.1A | 24,463647 | 120,82091 | 896,8 | 198,4 | -21,7 |  |
| CL19 | HLC040.1A | 24,463746 | 120,820762 | 878,7 | 177,2 | -26,1 |  |
| CL20 | HLC038.1A | 24,463989 | 120,820298 | 825,2 | 192,8 | -6,5 |  |
| CL21 | HLC037.1A | 24,464043 | 120,820258 | 819,4 | 182,6 | -41,4 |  |
| CL22 | HLC036.1A | 24,464088 | 120,820199 | 811,8 | 163,9 | -22,2 |  |
| CL23 | HLC035.1A | 24,464133 | 120,820041 | 795,1 | 177,7 | -12,0 |  |
| CL24 | HLC034.1A | 24,46416 | 120,819982 | 788,4 | 165,3 | -45,0 |  |
| CL25 | HLC033.1A | 24,464178 | 120,819933 | 783,2 | 176,9 | -33,2 |  |
| CL26 | HLC032.1A | 24,464214 | 120,819844 | 773,4 | 173,4 | -26,4 |  |
| CL27 | HLC031.1A | 24,464214 | 120,819706 | 760,3 | 171,7 | -38,6 |  |
| CL28 | HLC030.1A | 24,464358 | 120,819587 | 743,1 | 173,5 | -44,8 |  |
| CL29 | HLC029.1A | 24,464385 | 120,819597 | 743,1 | 204,0 | -12,0 |  |
| CL30 | HLC028.1A | 24,464394 | 120,819498 | 733,5 | 147,8 | -6,2 |  |
| CL31 | HLC027.1A | 24,464476 | 120,819469 | 727,4 | 177,4 | -31,2 |  |
| CL32 | HLC026.1A | 24,464448 | 120,819419 | 724,1 | 176,2 | -25,7 |  |
| CL33 | HLC025.1A | 24,464457 | 120,81935 | 717,1 | 178,5 | -22,5 |  |
| CL34 | HLC024.1A | 24,464512 | 120,81935 | 714,9 | 168,9 | 34,0 |  |
| CL35 | HLC023.1A | 24,464539 | 120,81933 | 712,0 | 162,9 | -13,6 |  |
| CL36 | HLC022.1A | 24,464647 | 120,819261 | 701,8 | 180,1 | -14,2 |  |
| CL37 | HLC062.1A | 24,465006 | 120,817801 | 549,8 | -9,3 | 18,1 |  |
| CL38 | HLC062.4A | no data | no data | 548,6 | -6,5 | 27,5 |  |
| CL39 | HLC062.10A | no data | no data | 546,6 | -2,8 | 20,5 |  |
| CL40 | HLC063.1A | no data | no data | 544,6 | -1,9 | 28,2 |  |
| CL41 | HLC063.5A | no data | no data | 543,6 | -1,2 | 17,6 |  |
| CL42 | HLC064.1A | 24,465151 | 120,817791 | 542,7 | 15,3 | 28,9 |  |
| CL43 | HLC064.4A | no data | no data | 539,6 | -5,1 | 21,8 |  |
| CL44 | HLC065.4A | no data | no data | 536,6 | 2,0 | 35,3 |  |
| CL45 | HLC080.3A | 24,465258 | 120,817297 | 491,8 | 1,4 | 27,4 |  |
| CL46 | HLC080.10A | no data | no data | 486,6 | 21,0 | 46,1 |  |
| CL47 | HLC081.1A | 24,465484 | 120,817267 | 480,4 | -4,5 | 16,2 |  |
| CL48 | HLC081.6A | no data | no data | 472,6 | -23,4 | 33,9 |  |
| CL49 | HLC021.1A | 24,467451 | 120,816387 | 321,8 | 195,3 | -25,0 |  |
| CL50 | HLC020.1A | no data | no data | 314,6 | 180,8 | -46,6 |  |
| CL51 | HLC019.1A | no data | no data | 306,6 | 180,0 | -20,4 |  |
| CL52 | HLC018.1A | no data | no data | 299,6 | 186,2 | -36,5 |  |
| CL53 | HLC017.1A | 24,467541 | 120,816150 | 291,5 | 191,2 | -31,7 |  |
| CL54 | HLC016.1A | 24,467514 | 120,815933 | 272,2 | 182,4 | -30,1 |  |
| CL55 | HLC015.1A | 24,467550 | 120,815814 | 258,4 | 189,5 | -32,4 |  |
| CL56 | HLC014.2A | no data | no data | 242,6 | 202,1 | -31,6 |  |
| CL57 | HLC013.1A | no data | no data | 234,6 | 182,9 | -36,0 |  |
| CL58 | HLC012.1A | no data | no data | 226,6 | 179,1 | -38,8 |  |
| CL59 | HLC011.1A | 24,467703 | 120,815459 | 219,4 | 188,8 | -43,3 |  |
| CL60 | HLC010.1A | 24,467784 | 120,815331 | 204,1 | 192,4 | -40,5 |  |
| CL61 | HLC009.1A | no data | no data | 195,6 | 189,4 | -35,4 |  |
| CL62 | HLC008.1A | no data | no data | 186,6 | 194,8 | -29,7 |  |
| CL63 | HLC007.1A | 24,467820 | 120,815064 | 178,1 | 186,5 | -35,3 |  |
| CL64 | HLC006.1A | no data | no data | 174,6 | 177,6 | -35,7 |  |
| CL65 | HLC005.1A | no data | no data | 171,6 | 181,9 | -35,0 |  |
| CL66 | HLC004.1A | no data | no data | 168,6 | 199,2 | -40,6 |  |
| CL67 | HLC003.2A | no data | no data | 163,6 | 197,6 | -41,0 |  |
| CL68 | HLC002.1A | no data | no data | 161,6 | 190,6 | -13,1 |  |
| CL69 | HLC001.1A | 24,467973 | 120,814926 | 158,8 | 203,7 | -32,6 |  |
| CL70 | HLC048.1A | 24,467982 | 120,814778 | 144,0 | 183,8 | -32,5 |  |
| CL71 | HLC049.1A | 24,468027 | 120,814679 | 132,9 | 174,1 | -37,1 | medium *Gephyrocapsa* |
| CL72 | HLC050.1A | 24,468225 | 120,814245 | 83,2 | 187,2 | -20,4 | medium *Gephyrocapsa* |
| CL73 | HLC051.1A | 24,468379 | 120,814047 | 58,6 | 213,0 | -52,5 | medium *Gephyrocapsa* |
| CL74 | HLC052.1A | 24,468469 | 120,813939 | 44,5 | 192,8 | -18,6 | medium *Gephyrocapsa* |
| CL75 | HLC053.1A | 24,468613 | 120,813781 | 23,7 | 210,8 | -35,6 | medium *Gephyrocapsa* |
| CL76 | HLC054.1A | 24,468775 | 120,813593 | -0,3 | 187,2 | -17,2 | medium *Gephyrocapsa* |

**Supplementary Information 2: Sedimentary Facies**

**
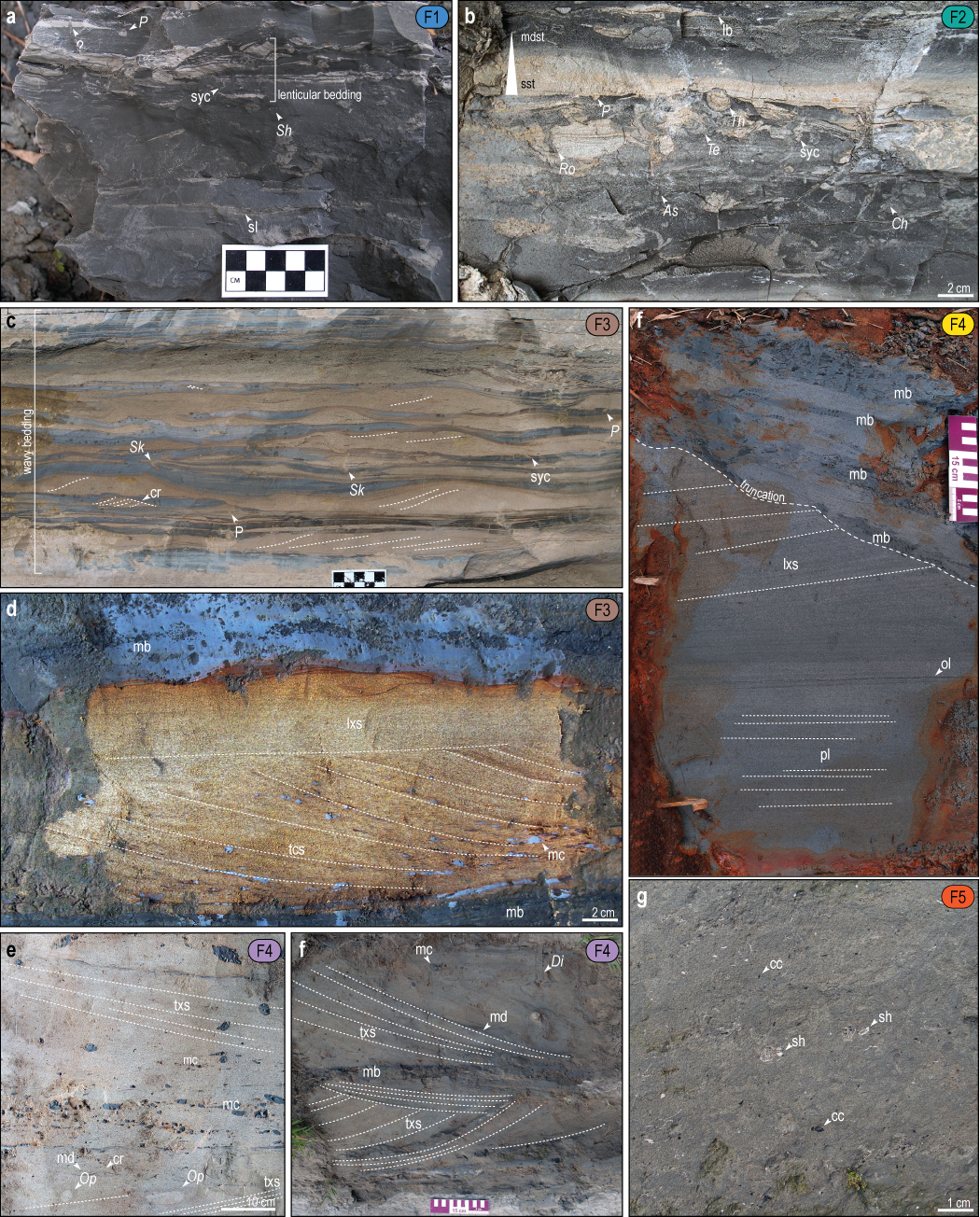
**

**Figure S5:** (A) Mudstone facies containing siltstone lenses and churned sandstone laminae (F1); (B) Mudstone-prone heterolithic facies showing a sharp-based, planar-laminated to normal-graded sandstone bed (F2); (C) Sandstone-prone heterolithic bed showing wavy bedding and current ripples (F3); (D) Unburrowed cross-bedded sandstone bed with mudstone rip up clasts overlain by a massive mudstone bed (F3); (E) Unburrowed sandstone bed exhibiting planar lamination and low-angle cross-stratification (F4). The bedform is top-truncated and overlain by a thick mudstone bed; (F) Mudstone-clast rich sandstone with planar lamination and trough cross-stratification (F4); (G) Trough cross-bedded sandstone with mudstone drapes and mudstone interbeds; (H) Highly bioturbated muddy sandstone (F5). *Pl: Planolites; Sh: Schaubcylindrichnus; Ro: Rosselia; As: Asterosoma; Te: Teichichnus; Th: Thalassinoides; Ch: Chondrites; Sk: Skolithos; Di: Diplocaterion*. sh: shell hash; cc: coal clast; mc: mud clast; mb: mudstone bed; ol: organic lamina; lxs: low-angle cross-stratification; pl: planar lamination; txs: trough cross-stratification; cr: current ripple; mdst: mudstone; sst: sandstone; syc: syneresis crack; sl: sandstone lamina; fb: flaser bedding; wb: wavy bedding; lb: lenticular bedding.

**Table S2:** Sedimentary facies description and interpretation from the lower part of the Cholan Formation along the Houlong River. Colors are consistent with the color code used in Fig. 3.

| **Facies** | | **Grain size** *main* (minor) | **BI (0-6); trace fossils** | **Description** | **Interpretation** | **Depositional environment and water depth*** |
| --- | --- | --- | --- | --- | --- | --- |
|  | F1: Massive to laminated mudstone | Clay (very fine to fine sand) | 0-1; *Skolithos, Schaubcylindrichnus, Planolites, Thalassinoides* | Massive to laminated mudstone beds. Medium gray to dark gray. Lenticular bedding, thin upper very fine to lower fine-grained sandstone laminae, local syneresis cracks | Low energy environment relatively, mud accumulation driven by suspension fallout and sporadic distal hyperpycnal discharge. Probable episodic salinity fluctuation. | Offshore intermittently affected by tropical cyclones  15 – 100 m |
|  | F2: Mudstone-dominated  heterolithic | *Clay - Silt* (very fine to fine sand) | 2-4; *Chondrites, Thalassinoides, Teichichnus, Rosselia, Asterosoma, Planolites* | Mudstone-prone heterolithic beds. Medium to light gray. Lenticular bedding, sharp-based, well-sorted fine-grained sandstone beds (locally normal grading) with low-angle cross-stratification, planar-lamination, trough cross-stratification, local hummocky cross-stratification and symmetrical cross lamination, and local syneresis cracks. Wood remains, disarticulated shells, shell hash | Relatively low energy environments sporadically disturbed by high-energy (top-down burrowed sandstone beds) event. Probable episodic salinity fluctuation and continental influence. |  |
|  | F3: Sandstone-dominated  heterolithic | *Upper very fine to upper fine sand* (clay; silt) | 0-2; *Skolithos*, *Diplocraterion, Planolites* | Sandstone-prone heterolithic beds. Interbedded upper very fine to upper fine-grained sandstone and mudstone. Mudstone are continuous to discontinuous, massive to laminated. Sandstone is well sorted. Light gray to light yellow. Wavy to flaser bedding, asymmetrical cross-lamination, trough cross-stratification, planar lamination, local symmetrical cross-lamination, structures mostly unidirectional, rare bidirectional, syneresis cracks. Coal clasts, organic lamination, mud drapes, wood remains, shell hash, disarticulated shells | Traction flow (current ripples and subaqueous dunes). Event beds deposition marked by unburrowed sandstones. Mudstone likely deposited as fluid mud. Coal clasts and organic material suggest continental influence. Salinity fluctuation. | Nearshore to proximal offshore intermittently affected by tropical cyclones  5 – 30 m |
|  | F4:  Cross-bedded sandstone | *Upper fine to lower medium sand* (clay; silt) | 0-1; *Ophiomorpha, Diplocraterion.* | Upper fine to lower medium-grained sandstone beds. Moderate to well sorted. Light yellow. Flat to slightly erosional lower contact. Planar lamination, low-angle cross-stratification, trough cross-stratification, asymmetrical cross-lamination, minor flaser bedding. Scours filled with structureless mudstone. Soft-sediment deformation, mudstone interbeds, mud drapes, rip up clasts | Cross-bedded sandstone deposited under tractive lower flow regime generating subaqueous dune with superimposed current ripples. Mudstone beds suggest fluid mud deposition. Low BI and soft sediment deformation point to a high-sedimentation rate. Mudstone-filled scours reflect internal erosion during energetic event followed by fluid mud deposition. |  |
|  | F5: Bioturbated  sandstone | *Upper very fine to lower fine sand* (clay; silt) | 6; Unidentifiable trace fossils | Upper very fine to lower fine-grained muddy sandstone beds. Dark gray. Poorly sorted. Massive. Abundant shell material (disarticulated shells, shell hash), coal clasts. | Reduced sedimentation rate with normal salinity condition and intense organism activities. | Nearshore to offshore  15 – 100 m |

*Depositional model developed for the Western Foreland Basin of Taiwan by Nagel et al. ^7^

**Supplementary Information 3: R code**

# R code by Christian Zeeden. 2021

# load libraries, which must be installed

library(readxl)

library(astrochron)

# read data (available in the Pangaea database)

Leg154_combined_benthic_isotopes <- read_excel("ref_datasets/global/Leg154_combined_benthic_isotopes.xlsx")

# optiona: view dataset

View(Leg154_combined_benthic_isotopes)

#extracting time and oxygen isotope data from file

bi <- cb(Leg154_combined_benthic_isotopes[,10], Leg154_combined_benthic_isotopes[,8])

# application of Taner filter

filter <- taner(linterp(bi), fhigh=1/10,roll=10^10)

# optional: export filter to text file

write.table(filter, 'filterBi_2020Dec17.txt')

**References**

1 Chen, P.-H. *et al.* Paleomagnetic and coccolith stratigraphy of Plio-Pleistocene shallow marine sediments, Chuhuangkeng, Miaoli. *Petroleum Geology of Taiwan* **14**, 219–239 (1977).

2 Kirschvink, J. L. The least-squares line and plane and the analysis of palaeomagnetic data. *Geophysical Journal of the Royal Astronomical Society* **62**, 699-718, doi:10.1111/j.1365-246X.1980.tb02601.x (1980).

3 Backman, J., Raffi, I., Rio, D., Fornaciari, E. & Pälike, H. Biozonation and biochronology of Miocene through Pleistocene calcareous nannofossils from low and middle latitudes. *Newsletters on Stratigraphy* **45**, 221-244 (2012).

4 Cohen, K. M. & Gibbard, P. L. Global chronostratigraphical correlation table for the last 2.7 million years, version 2019 QI-500. *Quaternary International* **500**, 20-31, <doi:https://doi.org/10.1016/j.quaint.2019.03.009> (2019).

5 Lee, J.-F. Geological Map of Taiwan - TungShih, Scale 1:50000, Central Geological Survey, MOEA, Taiwan. (2000).

6 Lattard, D., Engelmann, R., Kontny, A. & Sauerzapf, U. Curie temperatures of synthetic titanomagnetites in the Fe-Ti-O system: Effects of composition, crystal chemistry, and thermomagnetic methods. *Journal of Geophysical Research: Solid Earth* **111**, <doi:https://doi.org/10.1029/2006JB004591> (2006).

7 Nagel, S. *et al.* Sedimentology and foreland basin paleogeography during Taiwan arc continent collision. *Journal of Asian Earth Sciences* **62**, 180-204, doi:10.1016/j.jseaes.2012.09.001 (2013).
